# Reliable Measurement of Major, Minor, and Trace Elemental Nutrients

**DOI:** 10.6028/jres.093.074

**Published:** 1988-06-01

**Authors:** Milan Ihnat

**Affiliations:** Land Resource Research Centre, Agriculture Canada, Ottawa, Ontario K1A OC6, Canada

## 1. Introduction

A number of major, minor, and trace elements function as important nutrients in foodstuffs of crop and animal origin. These elements occur in a broad range of concentrations in an exceedingly vast array of foods available commercially and home-prepared. In disciplines such as regulatory compliance, product development, quality and safety, and research, reasonably reliable analytical information is mandatory for conclusions and decisions of impact. Although analytical scientists recognize the need for valid data, solid analytical information is at times elusive not only in the area of determination of chemical elements at low (trace) levels [1–5] but also when present as major constituents [6,7].

Of the many analytical techniques available for determination of inorganic nutrient constituents in biological matrices, those based on atomic spectrometry (AAS) are convenient and widely applied. The thrust of current research is to define impacts of a number of parameters on the performance of flame AAS (FAAS)-based methods of analysis of soil and biological materials to lead to reference methods for the measurement of major, minor and trace levels of elements of nutritional and toxicological pertinence. Some of these parameters as well as the excellent performance possible with well-applied versions of FAAS procedures are discussed.

## 2. High Reliability Flame Atomic Absorption Spectrometry

In spite of wide acceptance of the technique and the proliferation of published articles on its application, very few AAS-based methods have reached official method or reference method status [8], Requirements for analytical data of the highest reliability (high precision and accuracy) in the author’s research dealing with levels of elemental nutrients and toxicants in soils and biological tissues and with official and reference versions of procedures based on AAS have led to detailed investigations of a number of parameters bearing on method performance. Of the parameters listed in table 1, those studied thus far include sample decomposition, solution preparation, standard solution preparation, calibration approaches, instrumental parameters and measurement protocols.

**Figure 1:**
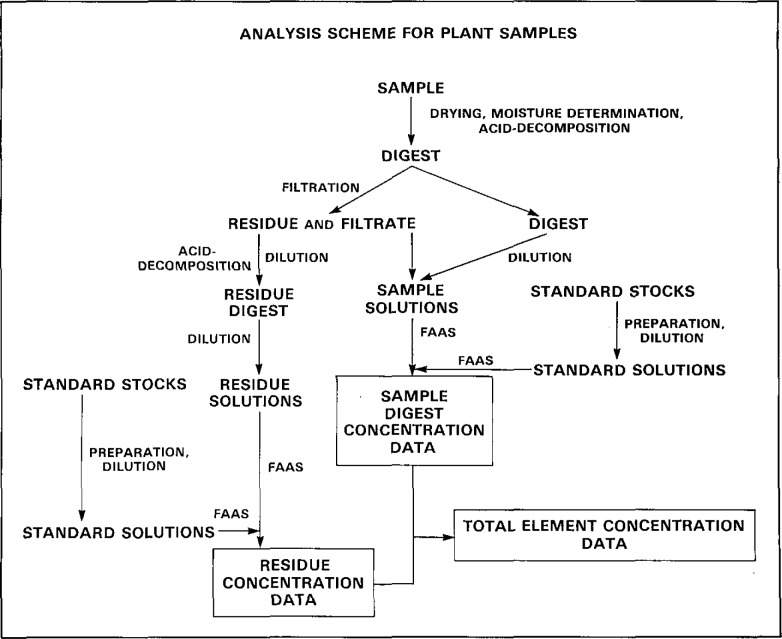
Scheme of analysis of plant samples by acid decomposition. Flame atomic absorption spectrometry for the determination of total elemental concentrations.

Experiments were conducted with four plant Standard Reference Materials as test materials, SRM 1570 Spinach, SRM 1571 Orchard Leaves, SRM 1573 Tomato Leaves, and SRM 1572 Citrus Leaves, from the National Bureau of Standards with the last product uncertified at the time of the study. Samples were dissolved with HNO_3_/HClO_4_ with further separate treatment of insoluble siliceous residues with HF for measurement of total concentrations of Na, K, Mg, Ca, Mn, Fe, Cu and Zn. Flame atomic absorption measurements were performed under optimized standard operating conditions. Measurement techniques were based on either bracketting of sample with appropriate standard solutions or second order calibration curves incorporating appropriate standard and procedural reagent blanks. Two types of working standard solutions were prepared with one set (regular standard) containing the eight analytes at concentrations required for analysis. The second set (matrix-matched) contained the analytes at approximate concentrations occurring in the plant materials being analyzed and, in addition, contained three other major constituents, Al, S and P present in the samples to match the analytes and matrices of the analytical samples, The scheme of analysis is depicted in figure 1.

Development and application of a high reliability version of an analytical procedure requires familiarity with all factors having a possible bearing on the precision and systematic errors of the overall procedure. The effect of calibration expressed as differences in elemental concentrations measured using regular and matrix-matched standard solutions is a function of element and sample type. Differences in concentrations ranged from 0.2% to 10.2% depending on element and matrix, indicating that for measurements of the highest reliability, systematic error arising from calibration can be reduced by using reasonable close matrix-matched standard solutions [9], Effects of the two sample decomposition procedures leading to acid-soluble and total concentrations showed that error in analytical data, without considering any contribution from the residue, ranged from 0.04% for Mg in spinach to 42% for Na in orchard leaves for the three materials certified at the time of the study [9,10]. For high reliability analysis, decomposition technique must be assessed and contributions of residues must be incorporated either by separate decompositions or one step procedures [10,11].

**Figure 2:**
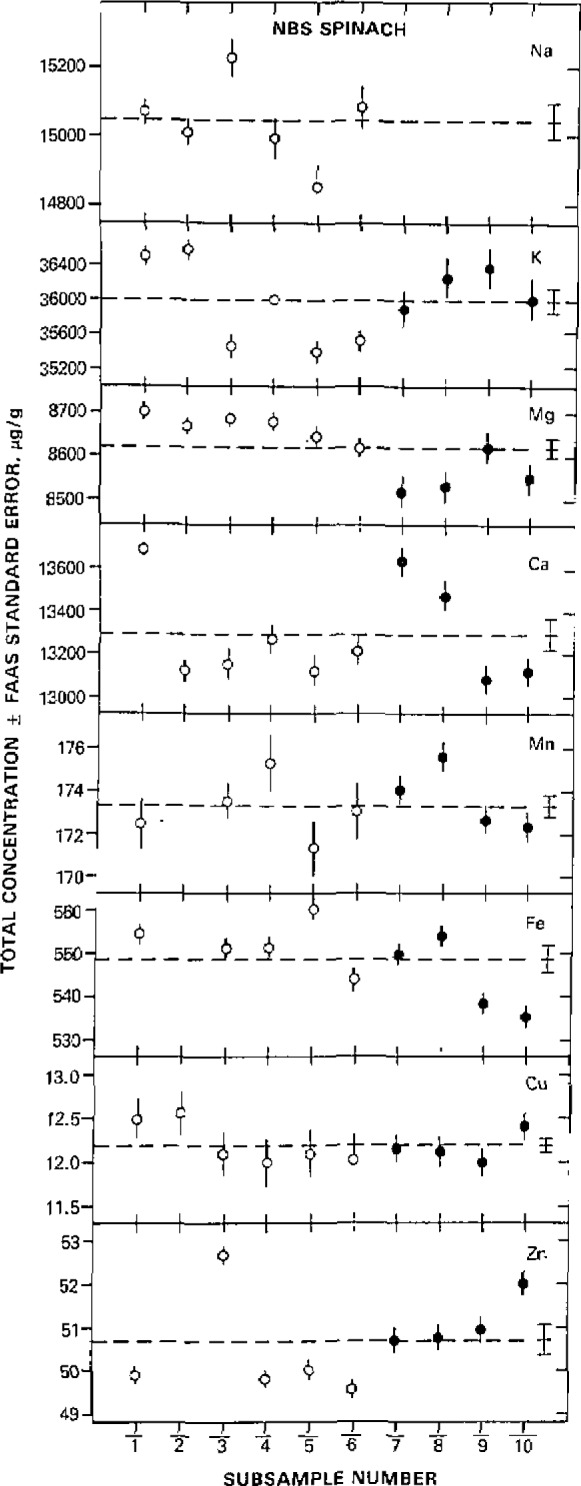
Total concentrations, with associated standard errors from the flame atomic absorption spectrometric step, of several major and minor elements in NBS SRM 1570 Spinach, as a function of subsample. Open and closed data points depict unfilteced and filtered solutions, respectively, used to measure acid-soluble concentrations; the data at extreme right are overall means ± standard error.

That meticulous attention to detail can lead to a high precision and high accuracy procedure is demonstrated by information in tables 2 and 3, and figure 2. Figure 2, depicting concentrations of eight elements in spinach determined by FAAS as a function of subsample, shows generally a tight scatter about the means. The means are compared in table 2 with certified values issued by NBS with excellent agreement evident among the two sets of data. A more striking demonstration of FAAS method performance in this work is provided by comparison (table 3) of results by this method with those from several other diverse methods of analysis applied during the certification work of NBS Citrus Leaves. Outstanding agreement can be observed between FAAS-generated data and those for other methods including INAA which importantly does not involve sample decomposition. Analytical results from this FAAS work were obtained before knowledge of the composition of the Citrus Leaves and in fact the data were used by NBS in the certification exercise. It is thus evident that consideration of appropriate details and careful work can lead to a truly high fidelity analytical procedure based on flame atomic spectrometry.

## Figures and Tables

**Table 1:** Some factors bearing on the reliable application of acid decomposition-flame atomic absorption spectrometry to the determination of major, minor and trace elements in biological materials

Sample decomposition and solution preparation
Volumetric ware verification, calibration, operational technique
Sample drying and/or moisture determination
Volatilization or retention losses in dry ashing or wet decomposition
Incomplete destruction of matrix; recovery and analysis of insoluble residue
Contamination from ashing acids, acids and vessels
Procedural and standard reagent blanks, non-identical performance of method with pure reagents and samples
Standard solutions, materials and preparative techniques
Sample and standard solution dilution schemes
Spectrometric measurement
Instrument optimization, performance characteristics and operational techniques
Physical and chemical interferences
Non-atomic absorption
Calibration solutions (single analyte, composite or matrix-matched)
Calibration technique (calibration curve, bracketting)
Data handling and interpretation
Recording, data entry and calculation
Calibration curve fitting and calculation techniques
Interpretation and evaluation (controls, statistical treatment, data presentation basis)
Overall
Analyst, specialist training and experience
Data quality control (accuracy verification by recovery testing and performance with appropriate reference materials)

**Table 2:** Concentrations of elements in NBS Spinach SRM 1570 determined by application of acid decomposition-flame atomic absorption spectrometry

Element	Total concentration, μg/g	
	This work ±SE^a^	Certified value
Na	1.504±0.005%	–
K	3.601±0.014%	3.56±0.03%^b^,^c^
Mg	8620±21	–
Ca	1.329±0.007%	1.35±0.03%^b^,^c^
Mn	173.3±0.5	165±6^c^
Fe	549±3	550±20^c^
Cu	12.1±0.1	12±2^c^
Zn	50.7±0.4	50±2^c^

a Typically 9 analyses; SE=standard error; SD = standard deviation.

b Values in weight percent.

c Uncertainty includes method imprecision, material inhomogeneity of entire SRM lot, and estimates of possible bias.

**Table 3:** Performance of high-precision, high-accuracy acid-decomposition flame atomic absorption spectrometry. Comparison with other methodologies/analysts for measurement of major and trace elements in Standard Reference Material 1572, Citrus Leaves

Method^a^	Concentration μg/g (mean±standard deviation)			
	K	Ca	Mn	Fe
FAAS	18140±160	31730±170	22.62±0.16	90.7±3.4
INAA	–	31710±60	22.6±0.14	89.3±4.3 91.7±0.8
IDMS	18180±70	–	–	–
FES	18230±100	30400±840	–	–
		31000±3000		
ICPAES	–	31600±400	22.1±1.1	90.1±3.7
POLAR	–	–	–	95.2±1.2
NBS	18200±600^b^	31500±1000^b^	23±2^b^	90±10^b^

a FAAS: Flame atomic absorption spectrometry (this work)

INAA: Instrumental neutron activation analysis

IDMS: Isotope dilution mass spectrometry

FES: Flame or plasma emission spectrometry

ICPAES: ICP atomic emission spectrometry

POLAR: Polarography

NBS: Certified values established by NBS after analyses completed.

b Uncertainties of NBS data are 95%/95% statistical tolerance intervals or based on judgment.
